# Effects of *Macrococcus caseolyticus* on the Volatile Flavor Substances of Chinese-Style Sausage

**DOI:** 10.3390/metabo15090570

**Published:** 2025-08-26

**Authors:** Yuanqing Gu, Xinya Chen, Jingjing Mao, Xin Nie, Chenglin Zhu, Qin Zou, Qiqi Luo, Yudi Zeng, Luca Laghi, Gianfranco Picone, Zhiping Zhao

**Affiliations:** 1 College of Food and Biological Engineering, Chengdu University, Chengdu 610106, China; 212024095135012@cdu.edu.cn (Y.G.); 212023095135006@cdu.edu.cn (X.C.); mjingjing2021@163.com (J.M.);; 2 College of Culinary and Food Science Engineering, Sichuan Tourism University, Chengdu 610100, China; 3 College of Pharmacy and Food, Southwest Minzu University, Chengdu 610041, China; 4 Department of Agricultural and Food Sciences, University of Bologna, 47521 Cesena, Italy; l.laghi@unibo.it (L.L.); gianfranco.picone@unibo.it (G.P.); 5 College of Chemical Engineering, Sichuan University of Science and Technology, Zigong 643000, China

**Keywords:** *Macrococcus caseolyticus*, sausage, physicochemical property, volatile flavor substances, gas chromatography–ion mobility spectrometry

## Abstract

Objectives: The primary objective of this study was to investigate the effects of *Macrococcus caseolyticus* isolated from Chinese bacon on the quality of Chinese-style sausages. Methods: The physicochemical properties and volatile flavor compounds (VOCs) of sausages inoculated with *M. caseolyticus* at different concentrations (10^5^, 10^6^, and 10^7^ CFU/g) were investigated. VOCs were detected using gas chromatography–ion mobility spectrometry (GC-IMS). Results: The sausages inoculated with *M. caseolyticus* showed progressive decreases in Aw, total volatile base nitrogen (TVB-N), malondialdehyde and carbonyl content during fermentation compared to the control sausage. A total of 90 VOCs were identified based on GC-IMS analysis, including 20 esters, 17 aldehydes, 22 alcohols, 12 ketones, 5 acids compounds, and 14 other compounds. *M. caseolyticus*-inoculated sausages exhibited elevated levels in alcohols and aldehydes, while the content of ketones was reduced compared to the control sausage. Multivariate statistical analysis indicated the significant differences in volatile flavor profiles among the sample and control sausages. Notably, seven VOCs in sausages, including 1-octen-3-ol, isoamyl alcohol, heptanal, hexanal, methyl 2-methylbutyrate, ethyl isovalerate and 2-pinene, were identified as the key aroma compounds (ROAV ≥ 1). Conclusions: The fermented sausages inoculated with different concentrations of *M. caseolyticus* exhibited significant differences in VOCs. This study provides the support for employing *M. caseolyticus* to improve the overall quality and flavor profile of Chinese-style sausage.

## 1. Introduction

Fermented sausage is a representative category of traditionally fermented meat products in China. It is produced by blending raw meat with various seasonings in specific proportions, which is followed by marination, curing, stuffing into casings, and subsequent air drying. Meat products contain not only substantial levels of high-quality protein but also essential micronutrients such as iron, selenium, magnesium, potassium, and vitamins. Sausage’s unique flavor profile and high nutritional value contribute to its widespread popularity among consumers. Previous studies have demonstrated that both microorganisms [1] and endogenous enzymes [2] play pivotal roles in the formation of VOCs during sausage fermentation. During the spontaneous fermentation, the microbial community in sausage primarily originates from raw materials and the surrounding environment with lactic acid bacteria, staphylococci, and yeasts as the dominant groups [3]. However, reliance on indigenous microbiota often leads to inconsistencies in product quality and flavor characteristics [4]. The application of exogenous starter cultures has been shown to improve standardization and ensure more consistent flavor development in fermented sausages [5].

Alcohols, aldehydes, ketones, esters and other substances constitute the principal VOCs in fermented sausages, which play substantial contributions to the overall flavor of meat products [6]. These substances are primarily formed through the oxidative degradation of macromolecules, including carbohydrates, proteins, and lipids [7]. Microorganisms play a central role in regulating these biochemical processes, and variations in the composition of the microbial community can lead to significant differences in both the sensory attributes and product quality of fermented sausages. Starter cultures employed for the production of fermented sausages primarily are lactic acid bacteria [8], yeasts [9] and staphylococci [10]. For instance, *Lactobacillus helveticus* IMAUJBH1 has been shown to effectively suppress lipid peroxidation, while it can also enhance the flavor profile of fermented mutton sausages [11]. A study demonstrated that *Debaryomyces hansenii* HRB1 not only effectively inhibited lipid and protein oxidation but also enhanced the aroma profiles and overall sensory quality of traditional northern Chinese dry-cured sausages [12]. Moreover, the combined application of *Pediococcus pentosaceus* and *Staphylococcus carnosus* has been shown to synergistically promote the formation of characteristic flavor compounds in fermented sausages [13].

*M. caseolyticus*, a member of the genus *Macrococcus* and a predecessor of the *Staphylococcus* genus [14], was originally isolated from cow milk [15]. Research has revealed that *M. caseolyticus* exhibits a substantial potential for generating diverse VOCs [16]. Additionally, *M. caseolyticus* has a positive effect on the flavor of milk and cheese by participating in the degradation of casein and the generation of aroma precursors, including small molecule peptides and free amino acids [17]. Currently, *S. carnosus* and *Staphylococcus xylosus* are predominantly used for quality improvement in traditional Chinese fermented meat products, while research on the application of *M. caseolyticus* in sausages is relatively rare.

In this study, one *M. caseolyticus* strain with promising protease activity and non-pathogenic was isolated from Chinese bacon and inoculated into Chinese-style sausages at varying concentrations (10^5^, 10^6^, and 10^7^ CFU/g). VOCs were investigated using GC-IMS. In addition, the physicochemical properties, total plate counts (TPCs) and total cocci counts of the sausages were analyzed. These findings provide a theoretical basis for the selection of starter cultures and the improvement of flavor and quality in Chinese-style sausages.

## 2. Materials and Methods

### 2.1. Preparation of Starter Cultures

*M. caseolyticus* was inoculated on Mannitol agar medium (Solarbio, Beijing, China) at 36 °C for 48 h. A single colony was inoculated in TSB liquid medium and grown overnight at 36 °C. The overnight grown cultures were inoculated into fresh TSB liquid medium until OD_600_ reached 0.60. Cells were harvested by centrifugation at 9600× *g* for 10 min at 4 °C (TGL-20M, Xiangyi Co., Ltd, Changsha, China). The cell pellets were washed twice with sterile saline (0.9% NaCl).

### 2.2. Sausage Preparation

Lean pork meat and fat were purchased from Goldkinn Foods (Sichuan Goldkinn Industry Group Co., Ltd, Suining, China). Lean pork meat from was cut into strips, while fat meat was cut into cubes. Fat meat and lean meat (3:7, *w*/*w*) were mixed with 2.5% salt (*w*/*w*) and cured. *M. caseolyticus* was then inoculated into the cured mixture at concentrations of 10^5^, 10^6^, and 10^7^ CFU/g, corresponding to the inoculated sausages labeled M5, M6, and M7, respectively. Sausages without inoculation of *M. caseolyticus* served as a control (CK). The mixture was filled into natural pig casings and naturally air dried for approximately 7 d. After air drying, the sausages were vacuum-packed and stored at room temperature for about 28 d.

### 2.3. Determination of Aw, pH Value, Total Plate Counts and Total Cocci Counts

The water activity was automatically detected with an water activity meter (HD-5D, Wuxi Huake Instrument Co., Ltd., Wuxi, China). Five grams of sausages were homogenized in 45 mL of sterile water, and the pH was measured using a digital pH meter (Testo 205, Detu instrument Co., Ltd., Shanghai, China). Total plate counts (TPCs) and total cocci counts were respectively determined by incubating samples on PCA agar and Mannitol agar (Aoboxing, Beijing, China) at 36 °C for 48 h. Samples were collected at 0, 3, 7, 14, 21, 28, and 35 d.

### 2.4. Determination of Total Volatile Basic Nitrogen (TVB-N) Content

The content of TVB-N in sausages was determined according to the National Standard of the People’s Republic of China GB 5009.228-2016 [18]. Ten grams of sausage was placed in a distillation tube and homogenized in 100 mL of deionized water for 30 min. One gram of magnesium oxide powder (Kelong Chemical Co., Ltd., Chengdu, China) was added to the distillation tube and immediately connected to the automatic Kjeldahl analyzer (KDN-19C, Shanghai Fiber Inspection Instrument Co., Ltd., Shanghai, China). Automatic Kjeldahl nitrogen determination was performed according to the manufacturer’s instructions. Finally, the resulting distillate was titrated, and the TVB-N content was calculated. Samples were collected at 0, 3, 7, 14, 21, 28, and 35 d.

### 2.5. Determination of Malondialdehyde Content, Carbonyl Content, and Sulfhydryl Content

The malondialdehyde (MDA) content in sausages was determined according to the National Standard of the People’s Republic of China GB 5009.181-2016 [19]. Five grams of sausage was added to a 100 mL conical flask, 50 mL of 75 mg/mL trichloroacetic acid solution (containing 1 mg/mL EDTA) (Kelong Chemical Co., Ltd., Chengdu, China) was added, the mixture was shaken thoroughly, and the flask was sealed with a cork stopper. The sealed flask was then incubated in a thermostatic shaker (SHA-B, Jinnan Instrument Manufacturing Co., Ltd., Changzhou, China) at 50 °C for 30 min. After cooling, the mixture was filtered. Five milliliters of filtrate were reacted with 5 mL of 2.88 mg/mL thiobarbituric acid (TBA) solution (Kelong Chemical Co., Ltd., Chengdu, China) in a water bath at 90 °C for 30 min. The absorbance value was determined at 532 nm using a spectrophotometer (T6 New Century, Puxi General Instrument Co., Ltd., Beijing, China). The carbonyl content and total sulfhydryl group content were determined according to the instructions of the kit (Solarbio, Beijing, China). Samples were collected at 0, 3, 7, 14, 21, 28, and 35 d.

### 2.6. Gas Chromatography–Ion Mobility Spectrometry (GC-IMS) Analysis

VOCs from sausages were analyzed at the end of storage (35 d) using a FlavourSpec^®^ GC-IMS system (Gesellschaft für Analytische Sensorsysteme mbH, Dortmund, Germany) equipped with a syringe and autosampler unit for headspace analysis, following the method described in our previous study [20]. After incubation at 40 °C for 15 min, 500 μL of headspace content was automatically injected using a heated syringe at 85 °C. Chromatographic separation was performed on an MXT-1 capillary column (15 m × 0.53 mm) at 60 °C. Nitrogen (N_2_, purity ≥ 99.999%) was used as the carrier gas. The flow rate program was as follows: 2 mL/min (initial, held for 2 min), increased to 10 mL/min for 2–5 min, to 15 mL/min for 5–15 min, to 50 mL/min for 15–20 min, and to 100 mL/min for 20–25 min. The drift gas was N_2_ (purity ≥ 99.999%) with a drift gas flow rate of 150 mL/min. Three parallel experiments were carried out for every sample.

### 2.7. Calculation of Relative Odor Activity Values (ROAVs)

The VOCs of ROAV ≥ 1 are considered to be key aroma compounds [21]. The relative odor activity value (ROAV) of flavor compounds in sausages was obtained using the following formula:

$$\mathrm{ROAV}\approx 100\times \frac{C_{i}}{C_{\max}}\times \frac{T_{\max}}{T_{i}}$$

where C_i_ represents the relative content of VOCs (%), and T_i_ represents the threshold of the VOCs (μg/kg). C_max_ and T_max_ represent the relative content (%) and threshold (μg/kg) of the VOCs that contribute the most to the overall flavor, respectively.

### 2.8. Statistical Analysis

Three independent batches of fermented sausages were prepared, and all measurements for the four groups were conducted in triplicate. All results have been expressed as mean values ± standard deviation. The physicochemical properties and microbiological analysis were analyzed using a two-way analysis of variance (ANOVA) in SPSS 26.0.1 (IBM, Armonk, NY, USA), which considered the concentrations of *M. caseolyticus*, fermentation times and their interactions as fixed effects, while each replicate was considered a random effect. The qualitative analysis of VOCs was performed using the VOCal software (version 0.4.03) integrated in the GC-IMS system based on retention time and drift time. Two-dimensional spectra and chromatographic fingerprints of VOCs were automatically generated by the Reporter (version 11.x) and Gallery Plot (version 1.1.0.2) modules within the GC-IMS system, respectively. GraphPad Prism 10.3.0 (GraphPad Software, San Diego, CA, USA) was used for Tree Diagram plotting, while principal component analysis (PCA) and partial least squares regression analysis (PLS) were performed using MetaboAnalyst 6.0 (https://www.metaboanalyst.ca, accessed on 2 June 2025). The VIP score plot was performed using SIMCA 14.1 (Umetrics, Umea, Sweden). The heatmap was generated using the tools on the BioinFormatics platform (http://www.bioinformatics.com.cn, accessed on 2 June 2025).

## 3. Results and Discussion

### 3.1. Subsection Physical and Chemical Evaluation

#### 3.1.1. Aw and pH Analysis

**Figure 1:**
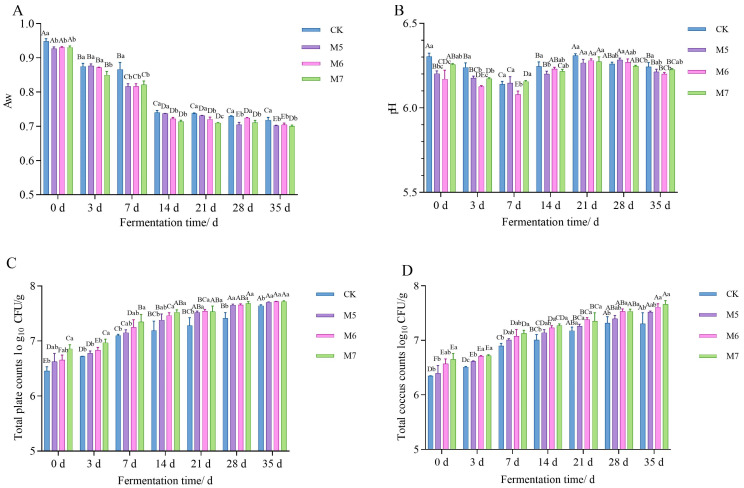
The effects of *M. caseolyticus* on the water activity (**A**), pH (**B**), total plate count (**C**) and total number of cocci (**D**) of the control and inoculated sausages during fermentation times. CK, M5, M6, and M7 represent the control sausage and sausages inoculated with the *M. caseolyticus* at the concentrations of 5, 6 and 7 log CFU/g, respectively. The different lowercase letters (a–c) indicate significant differences among the different treatments for the seam fermentation time, and different uppercase letters (A–E) indicate significant differences among the different fermentation times within a treatment group (*p* < 0.05).

As shown in Table 1, the fermentation time, *M. caseolyticus* concentrations, and their interaction significantly influenced the Aw and pH value of sausages (*p* < 0.05). The changes of Aw during fermentation are illustrated in Figure 1A. The Aw values of four groups decreased rapidly at the beginning of fermentation (*p* < 0.05). However, they slowly reduced after 14 days of fermentation. After 35 days of fermentation, the Aw for all the sausages, CK, M5, M6, and M7, decreased to 0.718, 0.702, 0.704, and 0.700, respectively. The results could be possibly attributed to the internal moisture migration and the evaporation of surface moisture during the drying process. At the end of fermentation (35 d), the Aw of the sausages inoculated with *M. caseolyticus* was significantly lower than that of the control group (*p* < 0.05). The decline in Aw possibly correlated with the microbial utilization of free water [22], suggesting that the Aw of the sausages was greatly diminished by *M. caseolyticus* fermentation.

As shown in Figure 1B, the pH values of all groups decreased during the first 7 days of fermentation, which was followed by an increase between 14 and 21 days. Notably, after 7 d of fermentation, the pH values of all samples were higher than those on 7 d. This trend is probably due to the continuous acid production by indigenous microorganisms during the early stages of fermentation, resulting in an initial pH reduction. As fermentation progressed, the microbial degradation of proteins generated peptides, amines, and other alkaline compounds, leading to a subsequent rise in pH [23]. On 7 d, the pH value of CK, M5, M6, and M7 reached the lowest at 6.14, 6.15, 6.08, and 6.16, respectively, with the pH of M6 group differing significantly from that of the other groups (*p* < 0.05). Additionally, the M6 group exhibited a lower pH at the end of storage, effectively inhibiting the growth of spoilage microorganisms and enhancing the storage stability of the fermented sausages.

#### 3.1.2. Microbiological Analysis

The fermentation time and *M. caseolyticus* concentrations significantly influenced the microorganisms (*p* < 0.05), although their interaction did not show a significant effect (*p* > 0.05), as shown in Table 1. At the beginning of the fermentation, the TPC values in the CK group (6.46 log CFU/g) were significantly lower than those in the M6 (6.66 log CFU/g) and M7 treatments (6.86 log CFU/g) (*p* < 0.05) (Figure 1C). Significant differences in TPC were observed between the CK group and the *M. caseolyticus* treatments, which could be related to the inoculation of starter cultures. With extension of fermentation time, both the TPC and the cocci counts gradually increased in all four sausage groups, reflecting the continuous microbial growth and proliferation in sausages during their maturation (Figure 1C,D). This growth trend of microorganisms was similar to the study reported by Liu et al. [24].

#### 3.1.3. TVB-N Content Analysis

**Figure 2:**
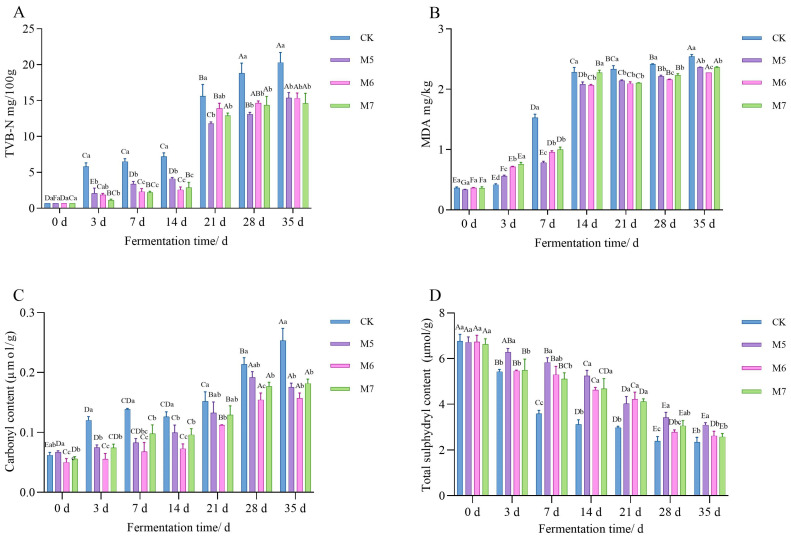
The effects of *M. caseolyticus* on the volatile basic nitrogen content (**A**), malondialdehyde content (**B**), carbonyl content (**C**) and total sulfhydryl content (**D**) of the control and inoculated sausages during fermentation times. CK, M5, M6, and M7 represent the control sausage and sausages inoculating the *M. caseolyticus* at the concentration of 5, 6 and 7 log CFU/g, respectively. Different lowercase letters (a–c) indicate significant differences among the different treatments for the seam fermentation time, and different uppercase letters (A–E) indicate significant differences among the different fermentation times within a treatment group (*p* < 0.05).

As shown in Figure 2A, the TVB-N content in the CK group was significantly higher than that in sausages inoculated with *M. caseolyticus* (*p* < 0.05). The increase in TVB-N content could be possibly attributed to protein degradation by enzymes and bacteria during the fermentation [25]. Initially, TVB-N values were approximately 0.70 mg/100 g, rising after 35 d of fermentation to 20.29 mg/100 g in the CK group, compared to 15.36, 15.32, and 14.63 mg/100 g in M5, M6, and M7 groups, respectively. As shown in Figure 1C,D, *M. caseolyticus* was capable of inhibiting the growth of other bacteria. Therefore, the inhibitory effect of *M. caseolyticus* on certain indigenous and spoilage microorganisms might be the main reason that the TVB-N of sausages inoculated with *M. caseolyticus* was lower than that of CK. These findings suggested that inoculation with the *M. caseolyticus* probably effectively suppressed spoilage in the fermented sausages. Similarly, previous research demonstrated that the inoculation of *S. xylosus* and *L. plantarum* in Harbin dry sausage effectively inhibited the TVB-N value [26].

#### 3.1.4. Antioxidant Capacity Analysis

As shown in Figure 2B, the MDA values for all four sausage groups gradually increased throughout the fermentation, which was likely due to the extended oxygen exposure that facilitated lipid oxidation. After 21 days fermentation, the MDA levels in the sausages inoculated with *M. caseolyticus* (M5, M6, and M7) were significantly lower compared to the CK group. At the end of ripening, the MDA values in the M5, M6, and M7 groups were 2.37, 2.27, and 2.36, respectively, and they differed significantly from the CK group (*p* < 0.05). These results suggested that inoculation with *M. caseolyticus* effectively inhibited lipid oxidation, potentially due to the secretion of specific antioxidant enzymes by coccus [27], which was consistent with a study by Chen et al [28].

As shown in Figure 2C, the carbonyl content increased markedly in all groups during fermentation with the CK group exhibiting the highest levels. Notably, the carbonyl content of the M6 group remained significantly lower than that of the CK group during the fermentation (*p* < 0.05). This indicated that the inoculation of *M. caseolyticus* could effectively inhibit protein oxidation in fermented sausages. Interestingly, the variations in total sulfhydryl content were inversely related to those of the carbonyl groups. According to Figure 2D, the total sulfhydryl content of the sausages gradually decreased during the fermentation with the proteins in M5, M6, and M7 showing higher total sulfhydryl contents compared to the CK group. The M5 group had the highest total sulfhydryl content at 35 days of fermentation (3.10 μmol/g), followed by the M6 group (2.62 μmol/g) and the M7 group (2.57 μmol/g), which indicated that inoculation with the starter could effectively reduce the total sulfhydryl content loss during sausage fermentation.

### 3.2. GC-IMS Analysis of the VOCs in Sausages

**Figure 3:**
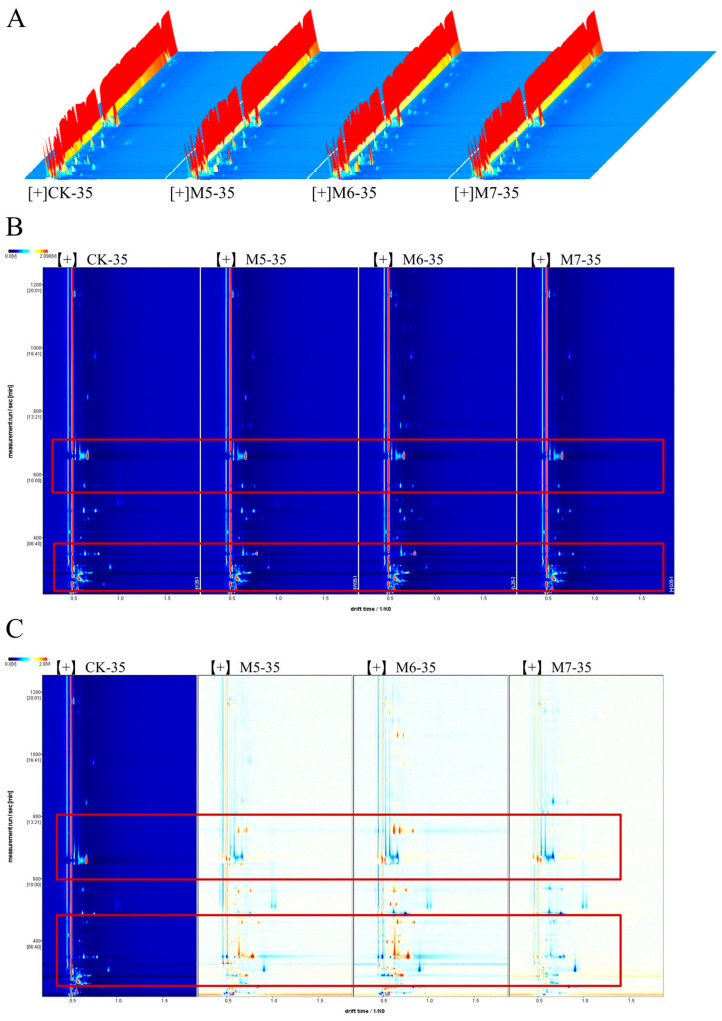
Analyses of the VOCs in the control and *M. caseolyticus*-fermented sausages detected by GC-IMS. (**A**) 3D topographic map; (**B**) 2D topographic map; (**C**) red and blue plots indicate increased and decreased aroma compounds in the *M. caseolyticus*-fermented sausages compared to the control sausage, respectively. CK, M5, M6, and M7 represent the control sausage and sausages inoculating the *M. caseolyticus* at the concentrations of 5, 6 and 7 log CFU/g, respectively.

During the initial fermentation phase, the sausages exhibited substantial variations in VOCs. With the extension of storage time, the VOCs in the sausages reached a relatively stable state. The differences in the VOCs in sausages inoculated with various concentrations of *M. caseolyticus* after 35-day fermentation were analyzed using GC-IMS, as shown in Figure 3. The results of topographic plots presented that the peak signal intensity of all sausages showed similar visualizations, whereas the signal intensity in each group indicated the differences (Figure 3A). The 2D plot of VOCs with different treatments was shown in Figure 3B. As the concentrations of *M. caseolyticus* increased, the content and profile of VOCs exhibited distinct variations. Most signals appeared in the retention time of 200–800 and the draft time of 1.0–2.0. In order to facilitate a clearer comparison of the variations in VOCs between samples, the CK group served as a reference. A white background indicated consistency in the detected VOCs, while red and blue colors indicated higher and lower values than that of the reference, respectively [29] (Figure 3C). In particular, the profile of the M6 group was significantly distinct from the other treatments, indicating a substantial increase in VOCs compared to the CK group.

**Figure 4:**
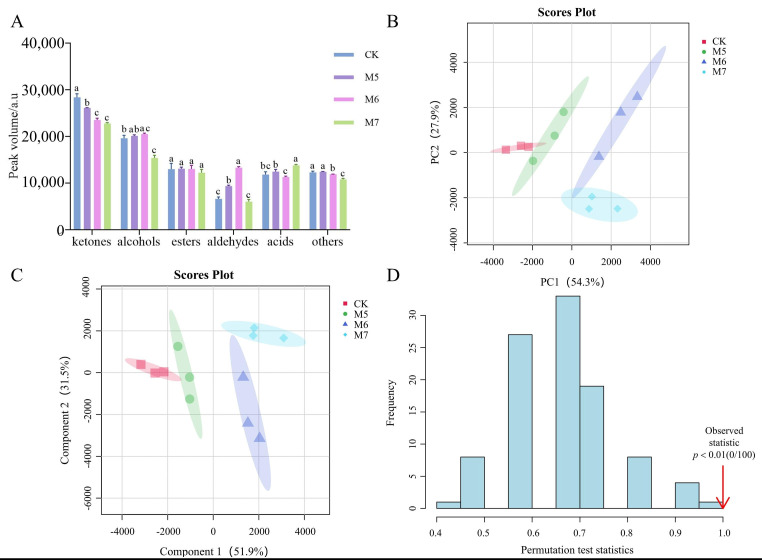
Analyses of the VOCs in the control and *M. caseolyticus*-fermented sausages detected by GC-IMS. (**A**) The peak volume of aroma compounds; (**B**) PCA score plot; (**C**) PLS-DA score; (**D**) Permutation test of the PLS-DA model. Different lowercase letters (a–c) indicate significant differences among the different treatments for the seam fermentation time. CK, M5, M6, and M7 represent the control sausage and sausages inoculating the *M. caseolyticus* at the concentrations of 5, 6 and 7 log CFU/g, respectively.

In order to further analyze the contents of various types of VOCs in sausages, the ionic peak volumes of VOCs in four groups of sausages were obtained (Table S1). A total of 90 VOCs were clearly characterized in the four samples by GC-IMS, comprising 20 esters, 17 aldehydes, 22 alcohols, 12 ketones, 5 acids, and 14 other compounds. Additionally, the peak volumes of various compound types in sausages were visualized in a bar chart, as shown in Figure 4A.

**Figure 5:**
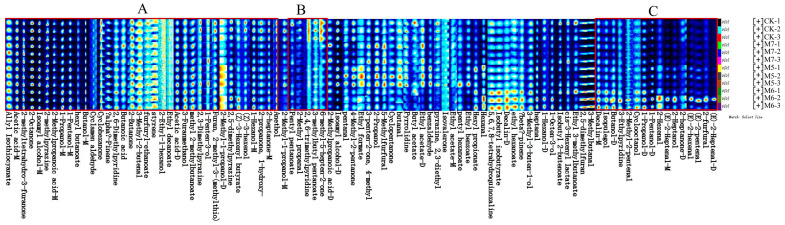
Fingerprint profiles of the VOCs in the control and *M. caseolyticus*-fermented sausages detected by GC-IMS. CK, M5, M6, and M7 represents the control sausage and sausages inoculating the *M. caseolyticus* at the concentrations of 5, 6 and 7 log CFU/g, respectively.

The fingerprints visually compared the changes in the VOCs in sausages with different treatments. As shown in Figure 5A, the VOCs with minimal variation in content across all sausage groups, including 2-octanone, ethyl hexanoate, cyclohexanone, 2-butanone, 2-ethyl-1-hexanol, ethyl decanoate and 3-furanmethanol, etc. In the CK group, the predominant VOCs were 2-methylpropanal, 3-methylbutyl pentanoate, pentyl pentanoate and 2,4,6-trimethylpyridine (Figure 5B). In contrast, the VOCs in the M6 group with high contents mainly included 3-methylbutanal, isobutyl isobutyrate, *cis*-3-hexenyl lactate, ethyl hexanoate, 2,5-dimethylfuran, 2-hexenal, heptanal and other volatile flavor compounds (Figure 5C).

Esters are mainly synthesized by non-enzyme-catalyzed esterification reactions of alcohols and acids and microbial enzyme catalysis. Owing to their low odor thresholds, esters can mask putrid flavor and play a crucial role in the flavor substances of fermented sausages [11]. There were no significant differences in the peak volumes of esters among the inoculated groups and the CK group (*p* > 0.05). Meanwhile, specific esters such as 1-hydroxy-2-propanone, ethyl hexanoate, isobutyl isobutyrate and hexyl propionate were more abundant in the M5 and M6 groups, contributing to the development of a pronounced fruity aroma in sausages.

In this study, ketones were identified as the predominant class of VOCs. Compounds such as 2-butanone, 2-octanone and 2-heptanone are known for their sweet, fruity, and floral aromas, which played a crucial role in the formation of sausage flavor [30]. Moreover, the contents of 2-heptanone-D and 2-heptanone-M were higher in the M6 group compared to the CK group. 2-Heptanone, derived from microbial β-oxidation, was primarily generated through *Staphylococcus* metabolism [31].

Alcohols are derived from the oxidation and degradation of lipids and have a pleasant fruity and floral aroma [32]. Notably, the content of alcohols in the M6 group was significantly higher than that of the CK group (*p* < 0.05), especially 1-octen-3-ol, 3-methyl-3-buten-1-ol, and 1-pentanol. Among these, 1-octen-3-ol with a distinctive mushroom aroma has been identified as one of the characteristic flavors in ham [33].

Aldehydes such as hexanal, nonanal, and heptanal are typical products generated by lipid autoxidation and enzymatic oxidation [8]. They have low flavor thresholds and strong volatility, contributing fruity, grassy, and floral aromas that significantly enhance the sensory profile of fermented sausages [34]. These compounds with moderate content can impart sausages with a positive flavor. It has reported that inoculation with *Lactobacillus* and *Staphylococcus* can increase aldehydes levels in sausages [1]. Additionally, the content of 2-methylpropanal with unfavorable odor was lower in the inoculated samples than that in the CK group, which could reduce the undesirable flavor of sausages. One study has shown that *Staphylococcus* sp. in fish sauce can enhance its characteristic aroma by metabolizing 2-methylpropanal [35].

As one of the important sources of flavor substances for fermented sausages, acids play an important role in the formation of esters. A total of five acids were detected in this study, including acetic acid and butyric acid, which were primarily produced through the microbial degradation of carbohydrates [36]. In addition, four pyrazine compounds were identified, which are desirable flavor substances commonly generated via the Maillard reaction and amino acid degradation [37]. The contents of 2,3-dimethylpyrazine, 2,5-dimethylpyrazine and 2-methylpyrazine were higher in the M6 group compared to the other groups, endowing a richer roasting and creamy aroma to sausages.

### 3.3. Multivariate Statistical Analysis of VOCs

To emphasize the differences in the VOCs profiles among sausages with different treatments, PCA was performed on four groups (Figure 4B). PC1 and PC2 each made contributions of 54.3% and 29.7%, respectively, bringing the total contribution to 84%. These findings suggested that the first two principal compounds can effectively reflect the information on volatile constituents in different groups. The distance between any two samples reflected their degree of variation. The CK and M5 groups clustered closely, suggesting similar VOC profiles, whereas M6 and M7 also grouped together but were positioned further from CK, indicating a distinct volatile composition influenced by the concentration of *M. caseolyticus*. The PLS revealed grouping patterns, consistent with the PCA results, as shown in Figure 4C. The PLS model was validated using a permutation test with 100 iterative cycles. As shown in Figure 4D, a statistically significant result (*p* < 0.01) demonstrated that the PLS model was robust and reliable with no evidence of overfitting.

**Figure 6:**
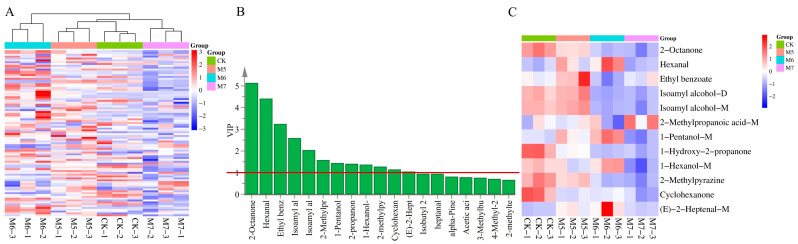
Clustering heatmap of VOCs in the control and *M. caseolyticus*-fermented sausages (**A**), VIP scores (**B**) and hierarchical clustering heatmap of the key aroma compounds (**C**). CK, M5, M6, and M7 represent the control sausage and sausages inoculating the *M. caseolyticus* at the concentration of 5, 6 and 7 log CFU/g, respectively.

The heatmap analysis (Figure 6A) yielded results consistent with the PCA score plot, which further highlighted the significant differences in the VOCs profiles among different treatment groups. Three parallel samples from the group were clustered together, indicating sufficient reproducibility between the samples. Notably, the CK and M5 group first clustered together, indicating that the M6 and M7 groups had a more different influence on flavor formation. However, the M6 and M7 groups were also not clustered in the same cluster, suggesting the differences in VOCs between them.

### 3.4. Analysis of the Differential VOCs in Sausages

Compounds exhibiting VIP > 1 make a significant contribution, and thus VIP > 1 was defined as a criterion for screening differential VOCs. Based on VIP > 1, a total of 12 VOCs were identified as differential markers across the four sausage groups (Table 2 and Figure 6B), including two ketones (2-octanone and cyclohexanone), two aldehydes (hexanal and (E)-2-Heptenal-M), two esters (ethyl benzoate and 1-hydroxy-2-propanone), four alcohols (isoamyl alcohol-D, isoamyl alcohol-M, 1-pentanol-M and 1-hexanol-M), one acid (2-methylpropanoic acid-M) and one other compound (2-methylpyrazine). These compounds were the differential compounds that distinguished the four groups of sausages.

As shown in Figure 6C, a hierarchical clustering analysis based on the 12 differential VOCs was performed to further explore the differences among the sausage samples. VOCs such as 2-octanone, 1-hydroxy-2-propanone, cyclohexanone and 2-methylpyrazine were higher in the CK group compared to the inoculated groups. This may be attributed to the regulatory effects of *M. caseolyticus* on metabolic pathways, potentially enhancing the catabolism or transformation of these compounds. Conversely, eight VOCs, including hexanal, ethyl benzoate, isoamyl alcohol-D, isoamyl alcohol-M, 2-methylpropanoic acid-M, 1-pentanol-M, 1-hexanol-M, and (E)-2-Heptenal-M, were higher in the inoculated sausages compared to the control. This difference was likely due to the ability of *M. caseolyticus* to promote the substantial accumulation of flavor precursors, thereby facilitating flavor development in fermented sausages [38].

Flavor perception is not only determined by the contents of VOCs but also related to their threshold values. ROAV is an index combining these two parameters to evaluate the contribution of VOCs. Compounds with ROAV ≥ 1 are considered key flavor substances, as they significantly influence the flavor of the product. In this study, seven key VOCs with ROAV > 1 were identified as the main contributors to the characteristic aroma of fermented sausages (Table 3). Among them, 1-octen-3-ol, methyl 2-methylbutyrate and ethyl isovalerate consistently exhibited ROAV > 1 across all treatment groups, which were the key VOCs in sausages. Specifically, 1-octen-3-ol imparted a mushroom-like aroma, methyl 2-methylbutyrate contributed fruity, green, and ether-fragrant aromas, while ethyl 3-methylbutanoate brought a sweet and sour smell. They all played important roles in the formation of sausage flavor. Additionally, the contributions of heptanal and hexanal were more pronounced in the M5 and M6 groups compared to the CK group, imparting the aroma of oil, fruits and vegetables.

## 4. Conclusions

This study investigated the effects of *M. caseolyticus* fermentation on the physicochemical properties and VOCs in Chinese-style sausage. The addition of *M. caseolyticus* reduced the Aw and pH levels of Chinese sausage at the end of storage, inhibited protein and lipid oxidation, reduced TVB-N levels, and promoted the formation of aldehydes and alcohols. A total of 90 VOCs were detected by GC-IMS. Significant volatile profile differences were observed among fermented sausages with differential *M. caseolyticus* inoculation levels. A total of 12 differential compounds were screened with VIP > 1, which could be used as a potential biomarker for distinguishing sausages with differential *M. caseolyticus* inoculation levels. Seven key VOCs by ROAV values (ROAV ≥ 1) were found to be the characteristic aroma of fermented sausages. Although this study proved the potential application of *M. caseolyticus* in sausage, the specific metabolic pathways mediated by *M. caseolyticus* and the mechanisms influencing the production of microbial metabolites remain unclear. In the future, metabolomics, metagenomics, and transcriptomics approaches will facilitate systematic investigation into the impact of *M. caseolyticus* on metabolite profiles and microbial community dynamics in fermented sausages.

## Figures and Tables

**Table 1:** The probability level (*p*-value) for an experimental main factor (fermentation time and *M. caseolyticus* concentrations) and their interaction effects on physical properties and microbiological analysis.

Experimental Factor and Interaction of Experimental Factors	Aw	pH	TPC	Coccus Count	TVB-N	MDA	Carbonyl	Sulfhydryl
Fermentation time	<0.05	<0.05	<0.05	<0.05	<0.05	<0.05	<0.05	<0.05
Concentrations	<0.05	<0.05	<0.05	<0.05	<0.05	<0.05	<0.05	<0.05
Fermentation time × Concentrations	<0.05	<0.05	0.121	0.917	<0.05	<0.05	<0.05	<0.05
Parameter trend	↓	↓	↑	↑	↓	↓	↓	↑

↑ indicates increased parameter levels with *M. caseolyticus*. supplementation compared to the CK group; ↓ denotes the opposite effect.

**Table 2:** The VIP values for differential VOCs in the control and sample sausages based on orthogonal partial least squares discriminant analysis.

	CAS	Flavor Substances	VIP	Aroma Descriptions
1	C111137	2-Octanone	5.14186	Cheese, flower
2	C66251	Hexanal	4.41696	Fruit, vegetable
3	C93890	Ethyl benzoate	3.24763	Flower, fruit
4	C123513	Isoamyl alcohol-D	2.59784	
5	C123513	Isoamyl alcohol-M	2.04378	Bitter, grease
6	C79312	2-Methylpropanoic acid-M	1.58308	Cream, cheese
7	C71410	1-Pentanol-M	1.44508	Camphor
8	C109944	1-Hydroxy-2-propanone	1.40796	Fruit
9	C111273	1-Hexanol-M	1.37159	Fruit
10	C109080	2-Methylpyrazine	1.27721	Roasted nuts
11	C108941	Cyclohexanone	1.14571	Mint
12	C18829555	(E)-2-Heptenal-M	1.05179	Grease, fruit

**Table 3:** The odor thresholds and ROAVs of volatile compounds in different fermented sausages.

Compounds	CAS	Retention Time/s	Threshold (μg/kg)	ROAV			
				CK	M5	M6	M7
1-Octen-3-ol	C3391864	1,138,229	1.50	1.02	1.33	1.20	1.04
Isoamyl alcohol	C123513	486,655	4.00	1.42	2.26	0.68	1.01
Heptanal	C111717	461,602	2.80	0.70	1.35	1.07	0.82
Hexanal	C66251	348,385	5.00	0.77	2.00	1.92	1.01
Methyl 2-methylbutyrate	C868575	311,674	0.25	2.63	4.43	3.13	3.74
Ethyl 3-methylbutanoate	C108645	252,942	0.01	100.00	100.00	100.00	100.00
Alpha-Pinene	C80568	29,052	4.60	0.61	1.16	0.83	0.92

## Data Availability

All datasets are available upon request from the authors.

## Supplementary Materials

The following supporting information can be downloaded at https://www.mdpi.com/article/10.3390/metabo15090570/s1, Table S1: Identification of volatile compounds in sausages.
