# Supplementary material for: Effects of Macrococcus caseolyticus on the Volatile Flavor Substances of Chinese-Style Sausage
Source: Metabolites. 2025 Aug 26;15(9):570. doi: 10.3390/metabo15090570 (PMC12471690; doi:10.3390/metabo15090570)
Supplement: Supplementary file 1 [file metabolites-15-00570-s001.zip › metabolites-3820030-Table S1.pdf]

Table S1. Identification of volatile compounds in sausages.

Volatile compounds	CAS	Molecular mass	RI	RT/s	Drift time/ms	Peak volume			
						CK	M5	M6	M7
Alcohols									
1-Pentanol-D	C71410	88,1	1228,2	566,567	12,535	337.24±18.83	478.96±103.73	669.40±114.72	248.76±61.34
1-Pentanol-M	C71410	88,1	1227,8	565,816	151,451	1970.19±59.83	2283.43±232.36	2699.19±185.92	1629.55±192.57
1-Penten-3-ol	C616251	86,1	1125,7	411,301	135,072	100.88±2.18	108.08±15.53	94.74±8.10	104.98±12.10
1-Octen-3-ol	C3391864	128,2	1451,9	1,138,229	115,342	715.64±33.74	633.68±38.05	826.17±173.72	510.26±86.66
2-Propanol	C67630	60,1	982,9	303,44	108,884	360.34±36.49	483.37±62.18	349.22±17.64	433.27±44.01
2-Heptanol	C543497	116,2	1315,2	756,349	138,221	119.73±15.35	210.71±47.71	357.84±202.72	153.70±25.25
2-Methyl-1-propanol-D	C78831	74,1	1058,3	349,678	117,335	120.62±2.53	132.21±10.84	92.01±11.37	108.74±16.97
2-Ethyl-1-hexanol	C104767	130,2	1492,1	1,250,472	180,015	467.65±21.31	405.34±30.32	407.51±39.72	430.17±27.91
3-Furanmethanol	C4412913	98,1	1675,1	1,761,773	11,016	1519.30±32.96	1510.06±40.10	1760.12±63.68	1506.00±80.48
3-Methyl-3-buten-1-ol	C763326	86,1	1229,1	568,335	11,739	422.30±27.64	428.83±19.43	509.69±28.47	357.42±17.05
2-Methyl-1-propanol-M	C78831	74,1	1057,1	348,878	136,976	741.25±25.07	661.33±101.02	467.12±90.58	562.40±16.28
1-Propanol-D	C71238	60,1	1003,8	313,444	111,217	259.16±20.04	276.38±5.47	542.51±44.08	207.19±28.33
1-Propanol-M	C71238	60,1	1002,5	312,559	125,432	1249.72±45.16	1294.09±19.26	1647.72±49.53	1143.48±69.37
Cyclooctanol	C696719	128,2	1131,6	418,744	157,283	87.45±5.50	92.65±6.07	135.19±14.45	94.96±19.11
(Z)-3-hexenol	C928961	100,2	1355,1	867,809	12,304	469.11±19.74	440.03±37.08	425.57±14.98	397.03±3.91
Isopulegol	C89792	154,3	1131,5	418,515	138,528	161.83±4.10	180.67±11.13	300.94±15.07	161.56±30.37
Isoamyl alcohol-D	C123513	88,1	1185,9	486,655	124,562	2647.17±205.37	2873.21±282.14	1250.84±69.65	1327.10±187.00
Isoamyl alcohol-M	C123513	88,1	1186,2	487,044	14,983	4366.27±115.86	4520.51±120.54	3280.38±76.87	3250.38±263.66
Butanol-D	C71363	74,1	1117,8	401,347	118,254	79.24±6.27	100.37±8.26	153.08±21.90	64.61±12.46
Butanol-M	C71363	74,1	1117,3	400,737	138,481	985.28±21.22	1039.02±9.82	1219.95±51.51	932.49±53.99
1-Hexanol-D	C111273	102,2	1347,6	846,976	132,582	369.09±16.54	348.77±19.88	357.11±48.78	281.73±29.22

Volatile compounds	CAS	Molecular mass	RI	RT/s	Drift time/ms	Peak volume			
						CK	M5	M6	M7
1-Hexanol-M	C111273	102,2	1347,5	846,485	164,504	2072.97±83.00	1771.31±198.45	2164.73±178.60	1312.58±206.53
Aldehydes									
(E)-2-Heptenal-D）	C18829555	112,2	1315,8	758,027	165,855	152.84±25.04	144.88±20.79	257.49±192.90	128.60±7.37
(E)-2-Heptenal-M	C18829555	112,2	1315,7	757,82	125,909	250.17±21.47	534.48±112.59	955.43±622.50	298.78±36.95
(E)-2-hexenal	C6728263	98,1	1204,8	519,921	118,172	158.29±9.87	206.11±45.22	332.04±159.90	174.42±8.38
2-Hexenal	C505577	98,1	849,4	254,532	117,507	84.61±1.68	110.80±28.69	223.12±83.15	86.65±11.09
2-Methyl-2-pentenal	C623369	98,1	1118,3	402,009	149,195	209.81±16.77	203.51±12.08	226.54±13.05	189.32±18.76
2-Methyl propanal	C78842	72,1	797,3	235,432	128,056	86.56±3.45	60.23±4.67	60.41±3.88	59.88±4.80
3-Methyl-2-butenal	C107868	84,1	1187,6	488,744	109,248	201.32±7.11	204.91±4.93	201.47±4.12	186.95±16.58
3-Methylbutanal	C590863	86,1	946,7	290,179	139,225	722.54±38.14	911.57±131.65	1175.83±219.22	658.53±99.50
5-Methylfurfural	C620020	110,1	961,2	295,48	112,864	590.93±41.38	708.08±123.33	506.54±89.69	763.02±67.67
Benzaldehyde	C100527	106,1	1509,7	1,299,723	115,238	341.69±4.51	508.27±15.14	382.51±47.32	334.28±23.67
Butanal	C123728	72,1	857,5	257,502	128,809	27.30±1.99	31.62±4.02	21.29±2.63	26.66±3.24
(E)-2-pentenal	C1576870	84,1	1114,2	396,907	110,601	101.52±5.23	151.75±27.44	227.48±123.42	122.51±2.10
Heptanal	C111717	114,2	1165,9	461,602	134,403	914.22±25.11	1201.79±179.75	1372.53±297.54	746.34±56.74
Hexanal	C66251	100,2	1056,4	348,385	155,974	1797.22±350.77	3167.56±1133.21	4400.20±1292.63	1644.50±336.29
2-Furfural	C98011	96,1	1425,5	1,064,649	133,265	210.85±19.37	217.17±33.44	336.87±193.35	164.90±22.67
Cyclamen aldehyde	C103957	190,3	1393,2	974,394	19,349	928.93±67.99	956.56±4.31	927.47±88.86	860.92±31.17
Pentanal	C110623	86,1	982,9	303,44	11,817	413.61±19.57	454.48±32.50	415.07±19.90	363.58±46.01
Esters									
(Z)-3-Hexenyl butyrate	C16491364	170,3	1452,7	1,140,451	143,575	777.44±20.27	647.15±14.14	746.88±14.76	657.37±26.52
Isobutyl 2-butenoate	C589662	142,2	989,5	305,852	180,453	1553.82±572.58	1228.00±335.28	1496.84±397.57	1257.97±381.11
Furfuryl-ethanoate	C623176	140,1	1493,5	1,254,534	141,799	390.88±12.99	401.77±44.98	406.52±22.00	384.17±30.75

Volatile compounds	CAS	Molecular mass	RI	RT/s	Drift time/ms	Peak volume			
						CK	M5	M6	M7
Methyl 2-methylbutanoate	C868575	116,2	1001,2	311,674	119,316	307.65±17.96	350.94±17.29	358.44±15.84	305.97±34.12
Ethyl benzoate	C93890	150,2	1667,5	1,740,548	125,991	2292.47±169.14	3338.76±776.62	1527.11±371.32	2258.66±345.61
Hexyl propionate	C2445763	158,2	1315,8	757,906	143,187	218.35±28.07	449.12±82.00	489.45±66.95	258.79±8.75
Hexyl butanoate	C2639636	172,3	1392,9	973,497	148,305	2333.84±55.89	2222.47±209.30	2585.28±385.52	2210.96±280.94
Ethyl decanoate	C110383	200,3	1446,0	1,121,859	225,602	1426.03±11.09	1642.61±341.12	1593.10±82.72	1425.02±68.52
Pentyl hexanoate	C540078	186,3	1490,7	1246,66	154,815	603.93±102.48	734.90±261.09	657.41±6.77	566.99±66.14
Ethyl formate	C109944	74,1	849,0	254,393	122,254	118.92±12.61	167.76±14.43	122.54±15.96	116.81±15.76
cis-3-Hexenyl lactate	C61931815	172,2	1200,3	510,81	197,199	795.13±250.13	664.53±148.11	774.01±171.34	725.98±271.11
Pentyl pentanoate	C2173560	172,3	1202,1	514,424	204,933	500.51±158.26	327.83±44.40	425.92±38.15	374.22±83.76
Ethyl acetate-D	C141786	88,1	850,6	254,946	109,821	182.11±25.83	234.82±20.81	109.15±10.46	223.11±43.83
Ethyl acetate-M	C141786	88,1	849,8	254,67	13,333	294.99±27.99	344.69±11.49	252.13±19.99	332.32±20.55
Butyl acetate	C123864	116,2	798,2	235,763	160,107	29.03±8.23	24.32±8.03	22.91±10.72	23.93±1.34
Isobutyl isobutyrate	C97858	144,2	1131,7	418,835	131,517	80.10±6.85	94.93±9.50	138.20±6.63	79.52±16.46
Allyl Isothiocyanate	C57067	99,2	867,5	261,163	108,917	216.98±3.46	246.22±6.05	239.56±6.13	240.56±5.63
Ethyl 3-methylbutanoate	C108645	130,2	845,1	252,942	167,326	467.92±196.54	318.17±124.88	455.34±205.15	326.22±104.61
Ethyl hexanoate	C123660	144,2	1001,4	311,807	134,089	46.35±0.96	51.73±3.87	64.78±3.94	37.83±4.44
3-Methylbutyl pentanoate	C2050091	172,3	1348,2	848,667	147,256	350.78±17.72	290.82±3.07	314.39±19.34	251.92±24.95
Ketones									
2-Butanone	C78933	72,1	862,6	259,375	106,142	287.79±7.02	304.33±11.04	287.99±6.21	275.23±24.39
2-heptanone-D	C110430	114,2	1161,3	455,905	126,128	58.93±3.12	63.22±12.94	82.71±21.59	37.61±7.14
2-Methyltetrahydro-3-	C3188009	100,1	1270,7	651,438	107,118	1935.96±28.94	2233.68±36.97	2292.59±22.51	2442.44±103.76

Volatile compounds	CAS	Molecular mass	RI	RT/s	Drift time/ms	Peak volume			
						CK	M5	M6	M7
furanone									
1-Hydroxy-2-propanone	C116096	74,1	1275,5	661,033	12,295	4044.96±227.96	3388.78±47.06	3087.90±32.34	3049.37±124.75
2-Octanone	C111137	128,2	1276,6	663,165	133,026	16901.54±479.93	15392.48±60.42	13387.01±312.41	12857.42±721.20
2-heptanone-M	C110430	114,2	1161,4	455,927	162,754	773.08±4.22	746.78±16.19	846.80±91.31	623.95±21.47
4-Methyl-2-pentanone	C108101	100,2	1057,3	348,98	147,888	694.31±33.09	905.36±35.83	584.32±11.31	382.42±40.89
3-penten-2-one, 4-methyl	C141797	98,1	1107,8	388,876	112,346	168.08±14.27	208.57±5.22	201.04±5.51	220.72±21.77
Isovalerone	C108838	142,2	1185,6	486,265	179,123	328.34±25.85	358.98±30.62	187.77±15.91	196.90±30.53
Cyclohexanone	C108941	98,1	1281,2	672,333	115,898	2669.14±201.31	2102.81±47.89	2145.80±36.64	1938.51±137.56
Cyclopentanone	C120923	84,1	1173,8	471,452	110,811	145.07±8.39	173.24±8.47	138.08±3.27	124.95±14.43
6-methyl-5-hepten-2-one	C110930	126,2	1329,8	797,243	11,733	363.55±22.90	229.08±10.28	203.41±8.77	219.29±25.56
Acids									
2-Methylpropanoic acid-D	C79312	88,1	1549,7	1,411,561	11,464	475.58±40.72	501.97±31.82	404.08±103.87	593.53±108.10
2-Methylpropanoic acid-M	C79312	88,1	1549,1	1,409,929	136,744	5129.76±384.01	5199.99±232.03	4808.10±772.02	5895.45±593.93
Butanoic acid	C107926	88,1	1603,5	1,561,768	116,582	1549.04±119.79	1590.03±114.02	1364.74±234.01	1713.29±134.01
Acetic acid-D	C64197	60,1	1464,3	1,172,957	105,495	487.88±20.19	566.22±30.83	539.20±26.74	536.37±38.43
Acetic acid-M	C64197	60,1	1463,8	1,171,539	116,764	4178.23±65.62	4576.91±107.46	4394.35±131.54	4559.14±157.21
Others									
2,3-Dimethylpyrazine	C5910894	108,1	1355,7	869,477	111,105	675.54±30.98	621.48±24.11	555.40±36.39	525.51±60.03
2,3-Diethylpyrazine	C15707241	136,2	1441,6	1,109,382	122,047	160.21±11.59	143.06±15.00	107.94±2.62	143.49±20.77
2,4,6-Trimethylpyridine	C108758	121,2	1381,6	941,982	115,119	262.91±7.56	211.56±13.78	216.28±10.95	187.03±8.74
2,5-Dimethylpyrazine	C123320	108,1	1336,0	814,439	111,207	743.13±49.44	657.30±61.75	626.26±27.70	471.19±19.33

Volatile compounds	CAS	Molecular mass	RI	RT/s	Drift time/ms	Peak volume			
						CK	M5	M6	M7
2,5-Dimethylfuran	C625865	96,1	943,0	288,814	136,292	842.02±26.50	895.48±69.49	1155.03±87.87	679.10±82.36
2,6-Dimethylpyridine	C108485	107,2	1248,8	607,733	10,957	182.51±7.94	216.54±6.85	212.68±18.00	204.47±17.81
2-Methyl-3-methylthiofuran	C63012975	128,2	1294,9	699,625	110,716	260.93±10.93	220.00±6.90	240.83±4.48	206.45±16.35
2-Methylpyrazine	C109080	94,1	1282,8	675,531	107,262	5966.07±147.35	5700.21±81.59	5118.69±132.52	4972.39±137.19
Alpha-Pinene	C80568	136,2	947,7	290,52	129,679	1314.87±51.49	1694.67±193.95	1759.99±192.70	1379.69±98,93
3-Ethylpyridine	C536787	107,2	1376,0	926,237	110,259	167.63±5.20	176.88±14.07	248.30±36.72	168.66±8.85
5,6,7,8-tetrahydroquinoxaline	C34413359	134,2	1228,4	567,008	120,144	215.37±10.99	206.99±9.74	268.17±1.53	171.62±18.46
Beta-pinene	C127913	136,2	1092,9	372,658	121,648	70.40±4.54	71.06±6.73	83.33±2.40	71.69±6.78
Styrene	C100425	104,2	1282,6	675,087	141,458	197.93±5.67	236.04±10.26	238.74±23.02	224.50±4.85
Anethol	C104461	148,2	1336,0	814,439	121,682	517.99±62.42	452.93±22.86	448.45±28.09	383.12±24.26
